# Supplementary material for: A space-based quantum gas laboratory at picokelvin energy scales
Source: Nat Commun. 2022 Dec 22;13:7889. doi: 10.1038/s41467-022-35274-6 (PMC9780313; doi:10.1038/s41467-022-35274-6)
Supplement: Supplementary file 1 — Supplementary Information [file 41467_2022_35274_MOESM1_ESM.pdf]

Supplementary Information

A space-based quantum gas laboratory at picokelvin energy scales

Naceur Gaaloul[1,⋆,†], Matthias Meister[2,†], Robin Corgier[1,3,#], Annie Pichery[1,3], Patrick Boegel[4], Waldemar Herr[5], Holger Ahlers[1,5], Eric Charron[3], Jason R. Williams[6], Robert J. Thompson[6], Wolfgang P. Schleich[2,4,7,8,9], Ernst M. Rasel[1], Nicholas P. Bigelow[10,⋆]

[1] Leibniz University Hannover, Institute of Quantum Optics, QUEST-Leibniz Research School, Hanover, Germany. [2] German Aerospace Center (DLR), Institute of Quantum Technologies, Ulm, Germany. [3] Université Paris-Saclay, CNRS, Institut des Sciences Moléculaires d'Orsay, F-91405 Orsay, France. [4] Institut für Quantenphysik and Center for Integrated Quantum Science and Technology (IQST), Ulm University, Ulm, Germany. [5] Deutsches Zentrum für Luft- und Raumfahrt e.V. (DLR), Institut für Satellitengeodäsie und Inertialsensorik, c/o Leibniz Universität Hannover, DLR-SI, Callinstraße 36, 30167 Hannover, Germany. [6] Jet Propulsion Laboratory, California Institute of Technology, Pasadena, CA, USA. [7] Hagler Institute for Advanced Study, Texas A&M University, College Station, TX, USA. [8] Texas A&M AgriLife Research, Texas A&M University, College Station, TX, USA. [9] Institute for Quantum Science and Engineering (IQSE), Department of Physics and Astronomy, Texas A&M University, College Station, TX, USA. [10] Department of Physics and Astronomy, University of Rochester, Rochester, NY 14627, USA.

[#] present address: LNE-SYRTE, Observatoire de Paris, Université PSL, CNRS, Sorbonne Université 61 avenue de l'Observatoire, 75014 Paris, France.

[⋆] Corresponding authors: gaaloul@iqo.uni-hannover.de, nicholas.bigelow@rochester.edu

[†] These authors contributed equally: Naceur Gaaloul, Matthias Meister

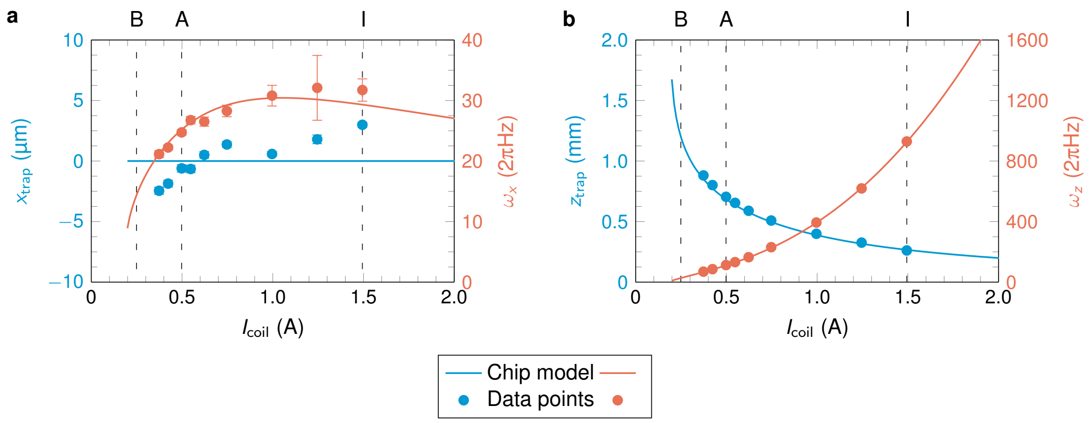

Supplementary Figure 1: **Calibration measurements and magnetic chip model**: Trap minima (blue) and trap frequencies (red) as a function of the current I_{coil} in the Helmholtz bias coils measured along the x-axis (a) and z-axis (b). Results from in-trap oscillation measurements (dots) are compared with the values predicted by the atom chip model (solid lines) based on solving the Biot-Savart law for the magnetic field generated by the chip wires and the bias coils of the CAL device. The error bars reflect the 1σ-confidence bounds of the fits. Overall the chip model agrees very well with the data points. Due to the geometry of the system the trap position in the x-direction is not expected to change as a function of the current I_{coil} which is supported by the measured deviation of less than 5 µm (a). The dashed vertical lines indicate the parameters for the initial trap I as well as the final trap of the transport ramps A and B, respectively. The calibrated chip model serves as an essential ingredient for designing efficient non-adiabatic transport ramps.

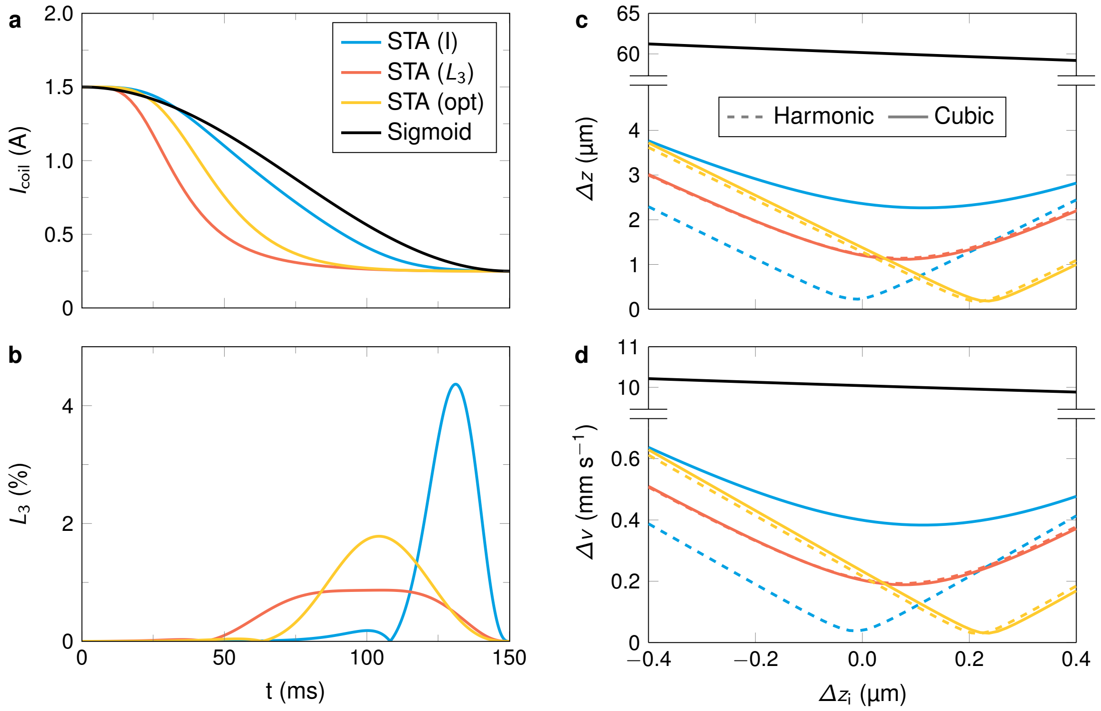

Supplementary Figure 2: **Robustness analysis of the transport ramps**: (a) Three STA theory ramps for the transport of the BEC. The smooth current ramp strategy (blue), the low-oscillation-amplitude ramp (red) and the trade-off ramp (yellow). A sigmoid ramp for an equivalent duration is shown in black. (b) Percentage of the cubic term out of the total potential explored by the center of mass of the BEC during the transport ramp for the three STA ramps. Residual offset (c) and velocity (d) in the final trap after the transport for different offset positions in the initial trap (see Fig. 1c). The continuous lines correspond to a potential including a cubic term whereas the dashed ones are for an idealized harmonic trap configuration. The different colors correspond to the different ramps shown in panel a. Here the smooth (blue) and lowest amplitude oscillation (red) ramps both lead to larger excitations in the final trap while the trade-off ramp (yellow) minimizes the COM excitations. The small offset to the ideal target state with vanishing residual oscillation amplitude and velocity is due to the limited amount of discretization steps of the current ramps and nicely coincides with the measured oscillation amplitude in the initial trap of 0.22 µm.

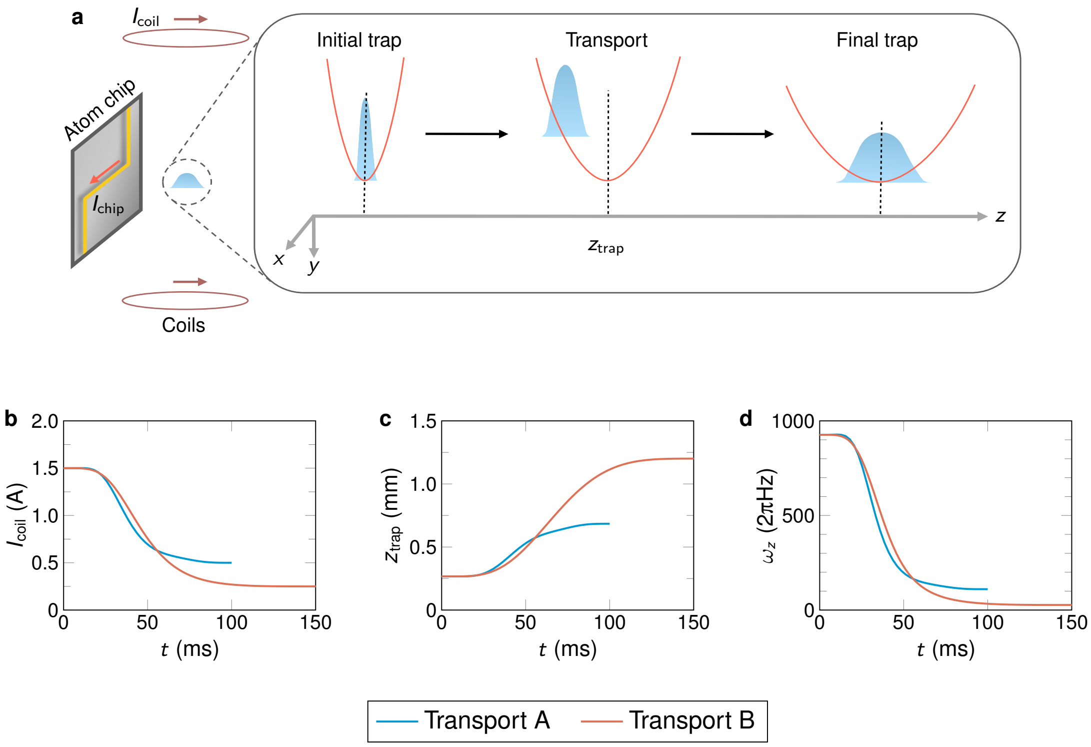

Supplementary Figure 3: **Details of the transport ramps**: (a) Scheme for the fast transport of a magnetically trapped ^{87}Rb BEC (blue wave packet) along the z-axis away from the atom chip while reducing the trap frequency ω_z over time. The trapping potential is formed by the magnetic fields generated by a current I_{chip} running through a Z-shaped wire on the atom chip and a pair of Helmholtz coils oriented along the y-axis with current I_{coil}. (b) Two different transport ramps A (B) are realized by lowering the current I_{coil} from initially 1.5 A to 0.5 (0.25) A within 100 (150) ms. (c) While lowering the current I_{coil} the minimum position z_{trap} of the magnetic trap is shifted away from the atom chip by a distance of 0.42 (0.93) mm for transport A (B), respectively. (d) Similarly, the trap frequency ω_z along the z-axis is reduced from $\omega_z = 2\pi \cdot 926$ Hz to $\omega_z = 2\pi \cdot 110$ (26.9) Hz.

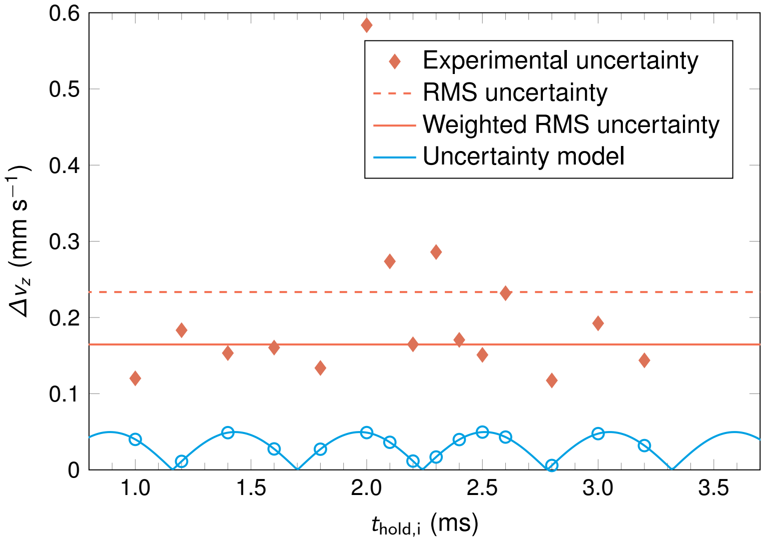

Supplementary Figure 4: **Analysis of BEC release uncertainty from trap B**: Measured error bars (red diamonds) of the release velocity after transport to trap B for different initial hold times $t_{\mathrm{hold,i}}$ as shown in Fig. 2b compared to the 1σ-confidence bounds (blue) obtained from the theoretical model. The measured uncertainties range from 117 µm s^{-1} to 286 µm s^{-1} with one exception for 2.0 ms hold time which is due to a low number of experimental shots (9) in contrast to the other timings (around 20 shots each). The root-mean-square (RMS) value of the experimental error bars (dashed red line) is given by 233 µm s^{-1} and decreases to 165 µm s^{-1} (solid red line) when the individual error bars are weighted with the 1σ-confidence bounds of the model. The weighted RMS uncertainty represents the overall quality of our release experiments.

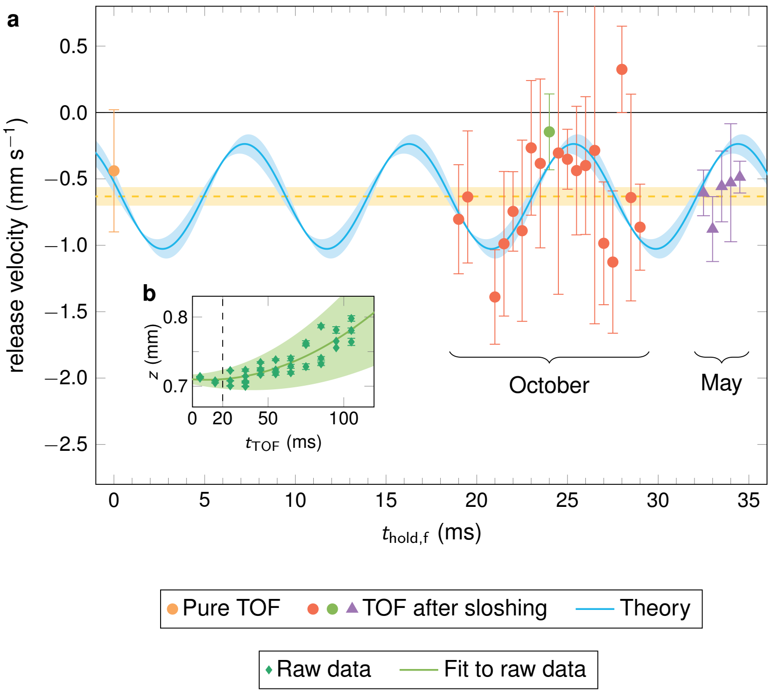

Supplementary Figure 5: **Long-term stability of the BEC release and preparation of atomic lensing**: (a) release velocity of a BEC transported to trap A with an optimal initial hold time $t_{\text{hold,i}}$ = 2.4 ms (see Fig. 2) and varying hold time $t_{\text{hold,f}}$ in the final trap. Data from May (purple triangles) is compared with a two-week campaign from October (orange, red and green dots) both showing good agreement with the theoretical model (blue line) although the average altitude of the ISS was increased by 7 km between both campaigns due to regular orbit adjustments. Here the velocity offset (yellow dashed line) Δv_A = -0.63 ± 0.07 mm s^{-1} is solely caused by the switch-off of the final trap (ω_z = $2\pi \cdot$ 110 Hz). The error bars and shaded areas show the 1σ-confidence bounds of the fits and the model, respectively. (b) Center-of-mass motion (green diamonds) of the BEC after release from the trap for the case $t_{\text{hold,f}}$ = 24 ms leading to a minimum release velocity v_z = -0.146 ± 0.286 mm s^{-1} obtained by fitting a parabola (solid line) to the data. In combination with the residual magnetic gradients the BEC has moved only 0.2 ± 5.9 µm within the first 20 ms of free expansion rendering it an ideal setup for subsequent atomic lensing. Green shaded areas show the 1σ-confidence bounds of the fits and error bars reflect the single-shot detection noise.

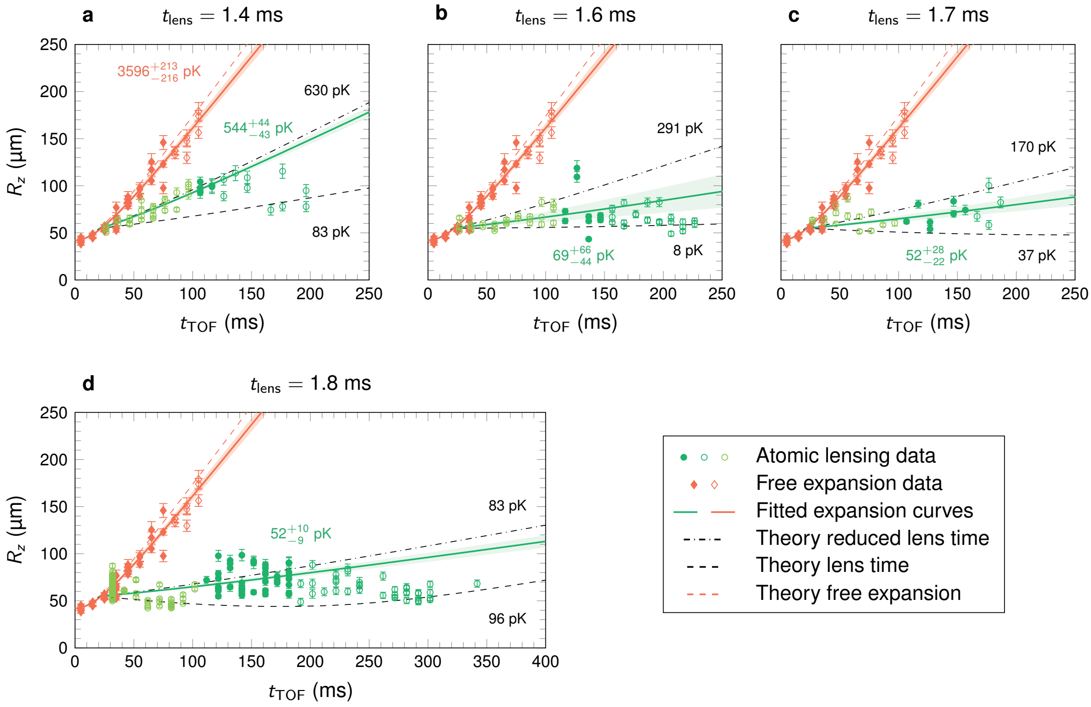

Supplementary Figure 6: **Control of the BEC expansion rate through delta-kick collimation with different lens times**: (a-d) Thomas-Fermi radius R_z of a free expanding BEC (red diamonds) released from trap A after a transport of 100 ms followed by 24 ms final hold time (see Supplementary Fig. 5) compared to a magnetically lensed BEC where the final trap was switched on again for a short time tlens (green dots) 20 ms after the release with rescaled angular frequencies (by a factor 1/4). The atomic lens removes kinetic energy from the system and allows engineering of different expansion rates. The expansion energy is determined by fitting theory curves (solid lines) to the data (filled dots) yielding values between 52 pK and 3.6 nK. Shaded areas show the 1σ-confidence bounds of the fits and error bars reflect the single-shot detection noise. Comparison with a realistic 3D simulation of the Gross-Pitaevskii equation (dashed lines) reveals that the effective lens is weaker than expected due to finite switching times and an upper bound of the expansion dynamics is given by a 0.4 ms shorter lens (dash-dotted lines) with the corresponding expansion energies given in black. Data shown by empty symbols are excluded from the fits due to split clouds at short times and low densities at long times (see Methods section).
